# Coronian Education: Perceptions of Educational Changes during the COVID-19 Pandemic in Arab Countries

**DOI:** 10.3390/ijerph19159223

**Published:** 2022-07-28

**Authors:** Abdulrahman Essa Al Lily, Ahmed Ali Alhazmi

**Affiliations:** 1 Department of Curriculum and Teaching Methods, College of Education, King Faisal University, Al Ahsa 31982, Saudi Arabia; aallily@kfu.edu.sa; 2 Department of Education, College of Education, Jazan University, Jazan 45142, Saudi Arabia

**Keywords:** Arab distance learning, Arabs, Arab distance education, coronavirus, COVID-19

## Abstract

This article tackles the question: To what domains did education go when it left school buildings due to the coronavirus pandemic? To answer this question, 1184 observations of online activity, 1132 observations of face-to-face activity, 118 focus groups and 1110 individual interviews were undertaken. In addition, 1290 witticisms were collected, utilising humour to inform research. Data analysis reveals the relocation of education to three domains: the domestic, digital and political. Its relocation to the **domestic domain** has meant increased familial responsibility, fuelling domestic tensions and conflicting with home-based distractions. Its relocation to the **digital domain** has involved reduced physical interaction, rituality, social merit, mobility and student health. Its relocation to the **political domain** has given rise to issues of participation and reshaped the power, institutional fabrication and societal support of education. The conclusion introduces the concept of “coronian education”—a hybrid of the domestic, digital and political domains. Whereas pre-coronian education was limited to a single domain, the school, coronian education is fragmented across three domains. Although coronian education research is feasible in the digital and political domains, it is challenging to conduct such research in the domestic domain, as an enquiry into domesticity entails invading the private spaces of homes.

## 1. Conceptual Framework

This 2021 study constructs a conceptual framework for education during the COVID-19 pandemic. It addresses the following perplexing research question: To what domains did education go after it left the school building? A detailed literature review was conducted, over a one-year period, on writings from early March 2020, establishing a databank of publications about education amid the COVID-19 crisis. A total of 1967 publications were discovered that possessed Clarivate-defined impact factors. Of those, 63% were about the transmission of education from the school building to the domestic, digital and political domains during the pandemic.

First, the domestic domain is defined in this manuscript as an area of private activity linked to the home. It is where actors customarily function based on family structures. Family life is generally associated with privacy, and access is usually limited to relatives. It is the second least researched setting, targeted by 19% of the reviewed works. This shortage is partly because educational researchers, as part of their formal graduate qualifications, do not receive specific training in researching domestic activity. Similarly, the field of educational research lacks methodologies for exposing homes to academic enquiry. The classification of this 19% of the reviewed works identified that they covered specific subjects. For instance, one study looked into the quality and usefulness of from-home schooling during the pandemic [1]. Another investigated such matters as mental health problems, nutritional security and physical inactivity experienced by the participants of from-home education and teaching [2]. Nusser delved into how those with special needs managed from-home education [3], whereas Agu probed into the economic status of households during from-home education [4]. One study also touched on ethnicity-related matters in families [5]. Another discussed the composition and content of from-home education curricula and examined learning and teaching using homemade tools [6]. Another issue that was explored was the imposition of more educational responsibility on households amid the pandemic [7]. Additionally, methodologies for conducting academic enquiries into education based at home were discussed [8].

Second, the digital domain is described as technical activity in which actors’ behaviours converge around an internet-based form. Here, the actors communicate only through technology-based channels. It is where digitalisation takes precedence over all other media. It is related to matters of digital concern. It is the most examined of the three settings, analysed by 78% of the reviewed writings. The availability of considerable funding for this domain spurs its popularity among researchers. Furthermore, digitalisation was the most noticeable feature of education during the pandemic. When categorising this 78% of the reviewed works, it was noted that this research covered specific topics. For example, Desai et al. deliberated on the effectiveness, quality and gains of internet-based education in both general and particular fields or subjects [9]. Pal and Vanijja thrashed out the issues of implementing online learning platforms (generic or in-house), augmented reality and educational simulation programmes during the pandemic [10]. One study presented positive experiences and best practices related to internet-based education [11]. Another critically reflected on specific teaching methods, curricula, educational courses and training programmes designed for internet-based education during the pandemic [12]. Germane to this was the evaluation of students’ digital skills, competencies and satisfaction in education during the crisis [13]. A further, hotly debated issue concerned online assessment and examinations [14]. Alongside this, the creation of models to predict the digital nature of education after the pandemic is over was also explored [15].

Third, the political domain is considered in this project as an area of everyday activity associated with political participation and thought, through which public opinion is formed. It is open and exposed to all, thereby becoming the business of everyone. Here, society collectively reflects on and shapes (and is shaped by) various configurations. It relates to matters of general interest. It is where social meaning and values are articulated, expressed and negotiated. It is the least researched domain, included in merely 2% of the reviewed publications. This scarcity is due to the departure of education from the school building to the political domain being a new phenomenon. Likewise, academics are unaccustomed to the inspection of education in the political sphere. After categorising this 2%, it was found that the studies covered limited topics. For example, one enquired into how education during the pandemic was intensively discussed and shaped through media channels and by journalists and social media activists [16]. Another topic was related to social media’s social, cultural, economic, political and educational roles in education during the pandemic [17]. This was in addition to capturing the various ways students and teachers utilised social media to learn and teach one another during the crisis [18].

The significance and unique contributions of this study can be summarised as follows. Whereas traditional education is limited to an individual school domain, this study seeks to examine how education during the pandemic has been fragmented across three domains (domestic, digital and political). The study also explores whether new actors have surfaced and catapulted education into "battlefields", as education has encroached on uncharted territories (domestic, digital and political domains). Moreover, it demonstrates how education’s departure from its cosy school domain may have deconstructed the historically persistent nature of this domain. It also discusses how education’s introduction to these new domains may require broadening the scopes and targets of research, complicating researchers’ missions. It also considers that, although education during the pandemic is easily researched in the political and digital domains, research in the domestic setting may be difficult given privacy laws.

The present study was, therefore, designed to be cross-national by covering several Arab nations: the United Arab Emirates, Jordan, Bahrain, Kuwait, Sudan, Oman, Qatar, Egypt, Oman and Saudi Arabia. The Arab domain was chosen because its culture plays a vital role in influencing education and all other aspects of its associates’ public and private lives. Arabs ordinarily interpret emerging issues (in this case, education during the pandemic) as cultural matters. To help an international audience understand the context of the research, what follows is background information about the norms in this context before the pandemic. Later in this article, the influence of the pandemic on these norms is shown. There used to be social and institutional resistance to online education. Schools traditionally prevented students from bringing mobile phones to school. Mothers played two roles in relation to children: an instructive, moral role (e.g., instructing children to act honestly) and an assistive, comforting role (e.g., making children feel supported and comforted). Grades were regarded as being based on hardship, not quality and outcome. Physical attendance was a critical factor in determining how Arabs valued matters. Arabs usually rated closeness above distancing. Students were not allowed to eat and snack during classes. Students and families had limited participation in shaping educational policies. It was traditionally preferable that women spend the least time possible outside the home. In some Arab regions, women never taught men; however, men could teach women, but only online. Although the students could see the teachers, the teachers could not see the students due to the culturally private nature of the female face. It had become permissible for the female voice to be heard in public as long as it was not recorded. In addition, boasting is a cultural practice in an Arab context. The Arab literature and online domains are awash with poems, novels and narratives wherein Arabs boast about their abilities, belongings and tribes. Arab culture entails such social norms as family solidarity and friend solidarity.

## 2. Methodology

This study puts education under a cultural microscope and focuses on the Arab context. The Arab domain was chosen because its culture plays a fundamental role in shaping education and all other aspects of its associates’ public and private lives. Arabs ordinarily interpret emerging issues (in this case, education during the pandemic) as cultural matters. Furthermore, many Arab countries were among the first globally to embrace and socially accept internet-based education during the pandemic, be it in the form of adult, special, prison, international or informal education. In other words, the top-down implementation of this type of education was accepted and adopted quickly by Arabs, with no explicit bottom-up resistance. This is partly because Arab society tends to sustain strong social contracts, whereby citizens expect the authorities to nurture and protect them and do what is best for them [19]. In addition, Arabs straightforwardly adopted this new form of education as it takes place at home. Arabs like to stay at home and regard the home as a domain that is sheltered against the wickedness of the outside world.

A research team was formed following the procedures explained in Table 1. We undertook 1184 observations of online activity and 1132 observations of face-to-face activity. The diverse range of activities observed included classes, field training sessions, extracurricular activities, examinations, workshops, laboratory activities, research projects, seminars, symposiums, conferences, managerial activities, parent councils, parties, counselling sessions, family gatherings and interviews with PhD and master’s applicants. Further data came from 118 focus groups and 1110 individual interviews. The strategy of maximum variation sampling was applied by approaching various groups, including students, teachers, teacher supervisors, headteachers, parents, directors of regional education departments, academics, college vice-deans and deans, and university vice-presidents and presidents. Approached groups not related to education included technicians, medical doctors, psychologists, domestic workers, businesspeople, media professionals, school security, salespersons, public transport drivers, librarians, food deliverers, social media activists and journalists. Groups related to the home were parents, grandparents, siblings, uncles, aunts, home visitors, live-in maids and babysitters. The participants still in education were diverse, ranging from pre-kindergarten, kindergarten, elementary, intermediate and secondary schools to undergraduate, postgraduate and post-doctorate programmes. Individuals associated with adult, prisoner, non-formal and international education settings were also included. Our diverse participants ranged from terminally ill to healthy, from illiterate to literate and from those with no formal education to those educated to the highest degree. They covered different socioeconomic classes, ages, genders, grades, disabilities and majors. They were tribal and non-tribal, urban and rural, and in the public, private and third sectors. Arabs as well as expatriates of Arab regions were approached.

Additionally, 1290 witticisms were collected and analysed, eccentrically utilising the participants’ humour to inform the research creatively. To this end, we assumed there was merit in understanding the social perceptions of education during the pandemic derived from an evaluation of the participants’ witticisms. Although witticisms suggest an absence of gravity, they are assumed to carry weighty meanings. They can reveal profound perceptions, especially since Arabs tend to manage crises through the use of witticisms. It is prevalent in Arab culture that individuals do not deal with each other directly out of politeness, nor do they express their opinions and points of view frankly. This results in difficulties in establishing Arab public opinion and temperament when directly asking people for their opinions in an interview or questionnaire. From this standpoint, this study investigated Arab public perceptions by resorting to indirect and roundabout methods (i.e., by analysing witticisms). We deemed the analysis of witticisms to be a practical methodology for identifying the social temperature of various phenomena and events.

Furthermore, the current study recorded and analysed 1163 initiatives established to facilitate education during the pandemic. Saudi Arabia appears at the top and forefront in terms of the number of initiatives introduced. These initiatives were launched by the public, private and third sectors (such as banks and charities) and by individuals (men and women, business leaders, royalty, religious figures and tribal sheikhs). They targeted students from limited-income families, orphans, those in quarantine or infected with COVID-19, refugees, individuals from war-torn cities or isolated villages, prisoners, adult learners, dropouts and the gifted. They were available either through their websites or social media sites. They were based locally, nationally, regionally or internationally. Some were reported, discussed and publicised in local and national newspapers and on television. They took the form of brochures, audio files and video clips. Due to the gender separation norms, the initiatives were, at times, directed at solely men or women.

**Figure 1:**
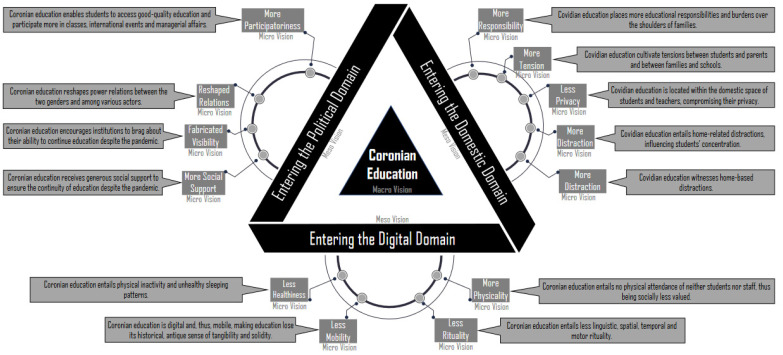
Data Collection and Analysis.

The data were analysed using NVivo, specifically utilising its mind-mapping feature. This analysis went through various stages, as illustrated in Figure 1. In the first stage (marks), each relevant statement by the participants was given a mark (a keyword or short phrase) that reflected its essential meaning. In the second phrase (micro-visions), marks of the same kind were grouped into micro-visions, which formed initial conceptualisations and a first step towards comprehension of the data. In the third stage (meso-visions), micro-visions of the same type were collated to constitute medium-level, sharper meso-visions, which entailed the next step in the data comprehension process. In the fourth stage (macro-visions), the meso-visions were finally assembled to create a coherent macro-vision (“coronian education”), constituting a complete comprehension of the data.

Ideally, natural language processing software (e.g., Repustate) would have been applied for the sentiment analysis of the data (i.e., fully automating the process by using computational clustering methods to identify micro-, meso- and macro-groups in the data) to eliminate potential bias in selecting terms and designing the framework. However, such software was previously employed in other Arabic-language mega projects without success. This can be attributed to a variety of reasons. One reason is that Arabic is a complex language with many varied dialects and with a high capacity for new word formations of rich semantic meaning. Hence, subjecting the data to natural language processing has, from previous experiences, resulted in inaccurate (and, actually, incorrect and misleading) findings. Based on these negative experiences, human intervention in the analysis of Arabic-language data is essential and unavoidable despite its limitations. Another reason in favour of the use of natural language processing would be to report the number of respondents in each category. The quantification of the data, however, was never the objective of the current ethnographical study, which rather strived to, qualitatively, build a conceptual framework and “make meanings” about the phenomenon under study. Nonetheless, assignments performed mostly manually have the potential to introduce bias into the study. In consideration of this, the authors, who have extensive training and practice in qualitative research, did their best to minimise any potential bias and to act according to the highest ethical standards.

Our analysis spotlighted various changes in education during the coronavirus pandemic, heralding the arrival of an uninvited, unorthodox education style: “coronian education”. This phrase is used for the remainder of this publication. This education concept was named after the virus because the pandemic has turned educational convention on its head. The phrase suggests how the virus became more than merely a health phenomenon or medical occasion: it remoulded education. Because Arabs generally like to name things using optimistic phrases, some participants were not in favour of this coined phrase given its association with the virus and, therefore, pessimism. Moreover, some Arab colleagues exhibited disapproval of the expression because it has not been accredited by any Western association, thus denying any authority in the West. In the following section, the findings are reported and referenced to Arab proverbs to examine the consistency or contradictions between the findings and the reviewed Arab proverbs. This is on account of proverbs functioning as reference points for Arabs.

## 3. Findings

### 3.1. Entering the Domestic Domain (Meso-Vision)

#### 3.1.1. Added Responsibilities (Micro-Vision)

Finding 1 was “shared responsibility”. Coronian education has mainly been family-centred because family members are predetermined to primarily educate their children. Due to the large numbers of children in Arab families, some families shared the task of educating the children and supervising them during their online classes among their members. Sometimes, a roster was created that established who supervised the children during the online lessons. Parents, older siblings, grandparents, uncles, aunts, live-in maids, neighbours and parents’ friends were all included on this roster. Mostly, mothers ended up taking responsibility for supervising their children. An Arab saying, *alomo madrasa* (“the mother is a school from which a child learns”), is typically used metaphorically to illustrate the influence of a mother over her child. However, with coronian education, this saying acquired a more literal meaning given that mothers were turned into "schools" responsible for their children’s formal education.

Finding 2 was “cheating mothers”. In coronian education, many mothers take the examinations for the children. Culturally speaking, *mahad beyasheel hamik khair omok* (no one carries others’ (school-related) concerns as much as a mother). Likewise, *elly omah fee aldaar qoresah haar* (one shall eat the best food (or get the best grades) if one’s mother is around). In one cartoon, a daughter instructs her mother: “Mom, study well, as I have an exam tomorrow”. Another witticism is “behind every correct answer is a mother”. These maternal, unethical interventions are, culturally speaking, problematic, considering that *ally yataealamah altifal meen omah yahfodah wayassomah* (what one learns from one’s mother (dishonesty) is implanted in one’s subconscious). Although a mother’s act of taking examinations for her children is a pure act of deception, Arabs do not perceive this as mothers cheating because, for Arabs, mothers are “divine” (interviewee).

Finding 3 was “taskless students”. Because parents were completing educational assignments and tasks on behalf of their children, a cartoon showed a child sitting in a resort and saying, “This is the best schooling ever: a year of holiday and passing is guaranteed”. It is said that *ealaa qader ahel aleazem taati aleazaem* (one’s excellent performance reflects one’s ambition), which is no longer accurate because students’ admirable performances reflect their parents’ ambitions instead. Another violated wisdom is *haq yador khaayr min bateel yaser* (truth that harms (children’s low performance) is better than falsehood (children’s fabricated performance) that brings joy).

#### 3.1.2. Fostered Tension (Micro-Vision)

Finding 1 was “parent–child conflict”. Coronian education has ushered in enhanced forms of disputes between parents and children. This was illustrated in a picture of a son doing homework: his father is behind him and sharply gazing at him while the son whispers, “the teacher is more merciful”. Another picture displayed an annoyed mother shouting, “I suggest to the Ministry of Education that they quarantine children and teachers in schools, and we just see them from a distance”. Because parents could attend students’ virtual classes at home, they realised that much of the blame that they used to direct to the teachers for their children’s failures was inaccurate and that the reasons for their children’s failures were the children themselves. Such realisations worsened conflicts between parents and children. Thus, the teaching staff exemplified the Arab proverb *rabun maluwm la dhanb lah* (the accused may be innocent).

Finding 2 was “school–parent clash”. Coronian education has entailed a boosted form of tension between teachers and parents. Coronian education required schools and families to work closely together, triggering more fighting. An audio clip revealed that a headteacher sent furious voice messages to mothers in their school’s *WhatsApp* group. Teachers regarded family members as “spies” (interviewee) who could secretly attend virtual classes from behind screens and report their pedagogical activities to higher authorities or social media. These teachers stressed their concern by saying that, in coronian classrooms, *aljudran laha adhan* (walls have spying ears). Another objection was that parents intervened considerably in virtual classrooms and undermined teachers’ control over classes.

Finding 3 was “child–teacher dispute”. There was a new form of conflict between children and teachers because coronian-educated students expected high grades for all their courses. Students believed that teachers should have considered that, in coronian times, anything that went wrong was “corona’s fault or teachers’ fault” (interviewee). To illustrate the point, a student wrote in a teacher evaluation form at the end of a semester that “given the unfortunate circumstances caused by (the) pandemic, the teacher needs to be flexible with grades, and all of us should get good grades”.

#### 3.1.3. Compromised Privacy (Micro-Vision)

Finding 1 concerned students’ privacy. In pre-coronian education, only teachers and educational supervisors could monitor students’ performance. However, under coronian education, more monitoring agents (basically, anyone granted access to students’ accounts) were authorised. Moreover, because classes were conducted at home, students’ partners, children, parents, siblings, grandparents, live-in maids and visitors to their homes could see and eavesdrop on the students’ performance during classes.

Finding 2 was about teachers’ privacy. In pre-coronian education, only headteachers and educational supervisors could observe teachers’ pedagogical activities, whereas coronian education allowed for increased numbers of observers and onlookers. One cartoon displayed many people watching an individual (labelled “teacher”) via multiple device screens, with the words “officials”, “student’s family” and “student’s whole tribe” under those watching. With profound frustration, one interviewee explained that, with teaching being broadcast from home, teachers’ partners, children, parents, siblings, grandparents, live-in maids and guests could listen to and, therefore, judge their performance, formal voice and ability to manage students.

#### 3.1.4. Enhanced Distraction (Micro-Vision)

Finding 1 was related to household size. An Arab house is conventionally beset by distractions. This is because an Arab family tends to include a large number of children, not to mention the grandparents, maids, siblings, aunts or uncles in residence. The noise level in Arab households tends to be “out of control” (interviewee). Seen in this light, a student’s ability to concentrate cannot be expected to be high.

Finding 2 was about visitations. Sudden and constant visits without prior arrangements constitute the norm in Arab culture. This prevailed throughout the pandemic and during the coronian school days at home. This represented distractions for the students in the households, especially since they tended to feel enthusiastic about visitors because of the opportunity to play with the guests’ children. Moreover, in Arab culture, when someone visits a household, all household members must welcome the visitor. If a family member does not show up and greet the visitor because they are busy with coronian schooling, this is considered a shameful act.

Finding 3 was “countered distraction”. Some serious students struggled with home-based distractions. To escape these distractions, some young students studied and attended online classes in restrooms or corridors. Adult students, however, could abscond to quiet coffee shops or the desert to study and participate in virtual lessons safely away from these distractions. This meant that cafés often became classrooms.

#### 3.1.5. Weakened Discipline (Micro-Vision)

Finding 1 was about individual discipline. Under coronian education, students were less likely to follow school disciplinary procedures because they were physically away from the physical punishments administered at school. Thus, *man amina aloqoba, assaae aladab* (one misbehaves if one knows of no surveillance (schools’ face-to-face surveillance)). Put differently, *etha ghab alqat, aleabb ya far* (during the absence of cats (teachers), mice (students) shall play).

Finding 2 had to do with collective discipline. Some students created clandestine WhatsApp groups (sometimes called, literally, “cheating groups”), wherein they shared answers to assignments and examinations among themselves. Some forced their “able” peers to contribute and share answers with these groups. These students may not have wanted to become involved in such misconduct but were pushed into it due to peer pressure. Students who did not contribute were stigmatised by their classmates who accused them of *mafik khair* (having no good for others).

Finding 3 was related to unfairness. Arabs subscribe to the belief that *fee alaimetehan, yukaram almaar aw yohan* (on the day of examinations, one is either honoured (with good grades) or instead humiliated (with bad grades)). However, in coronian education, students were honoured regardless because coronian education enabled students to cheat extensively. Consequently, this cheating damaged the long-standing maxim *man jada wajad, waman zarae hasad* (as you sow, so you will reap) because students could reap without sowing.

### 3.2. Entering the Digital Domain (Meso-Vision)

#### 3.2.1. Reduced Tangibility (Micro-Vision)

Finding 1 was about physical attendance. Coronian education has entailed no physical attendance of either the students or the staff, yet physical attendance is a critical factor in determining how Arabs value matters. To illustrate the point, important people were invited to be physically present at meetings, conferences, graduation ceremonies or exhibitions, whereas the rest of the guests were restricted to online attendance. To address this limitation, some educational institutions resorted to “blended presence” (interviewee), whereby some students were physically present, and others attended classes virtually. In this matter, physicality was associated with social worth and granted exclusively to prominent individuals. Phrased differently, coronian education used physicality to promote and preserve a sense of classism and social inequality.

Finding 2 concerned distancing. When education left the school building due to the pandemic, it became digital and lost its historical and traditional sense of tangibility. Once education became virtual, it became culturally and societally less valued. This consequence can be attributed to the socio-cultural perception that Arabs usually rate closeness above distancing. Thus, *albaeid meen aleayn baeid meen alqaleb* (far from the eye, far from the heart).

#### 3.2.2. Lessened Rituality (Micro-Vision)

Finding 1 was “spatial rituality”. Coronian education considerably diminished the conventional rituality of classrooms and schools—rituality that manifested in, for example, a set of equipment (e.g., boards, board pens, chairs, tables, projectors and bells).

Finding 2 was “temporal rituality”. Coronian education destabilised “divine temporal configurations” (interviewee). For example, in some schools, the historical lesson lengths, examination times and school day’s length were reconsidered and shortened in coronian education. This temporal efficiency chimes with the Arab adage *khayr alkalam ma qal wadal* (the best words are the fewest that hold the most meaning).

Finding 3 was “motor rituality”. The ritual code of conduct of pre-coronian education was no longer relevant, as coronian students (and teachers) came to possess very high levels of motor freedom, especially since they usually turned off their webcams. They could sit in the most comfortable way and, of course, eat and snack. Specific penalties were no longer applicable, such as moving an undisciplined student from one seat to another as a form of punishment. The lack of physicality in coronian education made students unreachable by traditional school disciplinary procedures, such as being prohibited from visiting bathrooms or standing out in the sun due to tardiness.

#### 3.2.3. Mobility (Micro-Vision)

Finding 1 concerned movability. Coronian education is considered “on-the-move education” (interviewee), as it enables schooling to take place anywhere. The crisis managed to bring the physical rigidity of education to a stop, making movability its bedrock.

Finding 2 was about spatial convenience. Although some pre-coronian schools traditionally prevented students from bringing mobile phones to school, in coronian education, mobile phones became the schools. Coronian education could occur in one’s most convenient space; hence, coronian students attended classes in unusual circumstances, such as being ensconced in desert tents or engaged in alternative activities such as herding camels, attending funerals or watching live camel racing. They participated in online classes on trains, cars, planes, buses and on the street. They also attended lessons while in sports clubs, parks, cafés, restaurants, farms, hospitals and clinics. 

#### 3.2.4. Unhealthiness (Micro-Vision)

Finding 1 was “inactivity”. Physical activity in pre-coronian education was represented in the commute to school, sports classes or group exercises performed during the morning assembly. In coronian education, however, physical activity became minimal because from-home education did not allow learners to practise their previous sports. Accordingly, coronian students suffered from health-related issues, such as obesity and spine problems, due to inactivity.

### 3.3. Entering the Political Domain (Meso-Vision)

#### 3.3.1. Participatoriness (Micro-Vision)

Finding 1 was “managerial participation”. Coronian students, parents and teachers turned to social media to ensure their participation in decision making and exert pressure on school management. Social media housed social campaigns and bottom-up e-votes over coronian school procedures and regulations. Such campaigns did not exist in pre-coronian education.

#### 3.3.2. Reshaped Relations (Micro-Vision)

Finding 1 was “reshaped stakeholders”. Although the pandemic prompted disasters for some, it opened a world of possibilities for others. As Arabs say, *masaebo qawmen enda qawmen fawaedo* (people’s misfortunes are benefits for others). For example, during the pandemic, government educational institutions saved on electricity, water, maintenance and computers. These expenses were transferred to households due to the from-home nature of coronian education. That said, families also saved on costs related to school clothing, supplies and meals. These expenses are exceptionally high for Arab households given the frequently large number of children in a family.

Finding 2 was “gender-based contention”. Some women reported disliking coronian education because it helped place women (namely, students and staff) back in their conventionally “rightful” place, considering that it is traditionally preferable that women spend the least time possible outside the home.

Finding 3 was “the female face”. In some Arab regions, women never taught men in pre-coronian education. However, men could teach women, but only online. Although the students could see the teachers, the teachers could not see the students due to the culturally private nature of the female face. In coronian education, male teachers found online teaching without seeing the students’ faces more accessible than female teachers because they were accustomed to this filtering in pre-coronian education. Similarly, female students found it more accessible than male students to be taught online because they were used to this methodology in pre-coronian education.

Finding 4 was “the female voice”. Before the pandemic, it had become acceptable in some Arab regions for the female voice to be heard in public as long as it was not recorded. That said, coronian education made recording the voices of female teachers, lecturers or trainers (and sharing them via social media) more possible, practised and tolerated. This is an example of how coronian education has facilitated (or has been facilitated by) some recent top-down reforms being initiated by some Arab authorities to reconfigure women-related communal norms and improve female citizens’ social status.

#### 3.3.3. Institutional Fabrication (Micro-Vision)

Finding 1 was related to awards. Many Arab institutions dedicated their energies, resources and efforts to win local, regional and international awards for the effective management of coronian education. This is because they wanted to brag about their ability to achieve the continuity of education despite the circumstances of the global pandemic. They regarded coronian education as an opportunity to show off globally and improve their international reputations. They were preoccupied with over-marketing their achievements; thus, *yaemal min alhubat quba* (making a mountain out of a molehill). This does not come as a surprise, as boasting is a cultural practice in an Arab context. The Arab literature and online domains are awash with poems, novels and narratives wherein Arabs boast about their abilities, belongings and tribes.

Finding 2 was about statistics. Institutions applied numbers and statistics to show their ability to continuously provide education despite the pandemic. Many Arab institutions obligated their employees to write daily reports on their teaching activities and produce periodic statistical reports, which the institutions published on social networking sites as sources of pride. Many faculty members were sceptical of the accuracy of the information provided in these reports because they considered it an unjustified additional burden on them. Hence, many such reports did not reflect reality, and thus, the learning outcomes were limited. Many institutions made a considerable effort to produce these inaccurate reports in an impressive and professional manner using infographics, supporting the Arabic proverb *min bara allah allah, wameen dakhil yaelamoh allah* (from the outside, it is impressive, and from the inside, only God knows).

#### 3.3.4. Social Support (Micro-Vision)

Finding 1 was “social assistance”. As many as 1163 collaborative social initiatives were taken to ease the experience of coronian education. This generous social support was provided because the educational experience was exposed to more challenges and hindrances than ever before during the pandemic.

Finding 2 was “materialistic givingness”. Individuals, groups and organisations gave money, devices and access to the internet to those students who needed them to access coronian education. Some utilised their *zakat* (a form of alms) to purchase equipment for the coronian education of poor students. One interviewee remarked, “I have given TVs as zakat for the poor, in the hope that God will reward me for good deeds; I would have never thought that TVs could be considered religious charity”. In this regard, coronian education has reshaped the social conception of what can be considered religious.

Finding 3 was “emotional givingness”. First, efforts were put into spreading a sense of positivity during coronian education. These included sharing positive maxims via social media. One such maxim is *aishetadi ya azma tanfarij’* (as much as a crisis becomes severe, as much as it eventually breaks loose). Another aphorism is *forajat wakonto azonoha la toferajo* (crises *do* break loose, even though one may think they never will). Second, initiatives were given inspirational slogans. Examples included “support me so that I may learn”, “we are with you”, “together to combat the coronavirus”, “let us work together”, “their education must not stop”, “let us learn”, “my home is my school” and “we are all family”. Third, relatives and friends relied on such social norms as family solidarity and friend solidarity to help one another with coronian education, echoing the proverb *alsadeq waqat aldee* (true friends reveal themselves during difficult times).

## 4. Conclusions

This article gave centre stage to the rise of coronian education. The main finding was that coronian education co-exists in three domains: the domestic, digital and political. The implication is that, although education in its pre-coronian phase was limited to one individual domain (schools), in the coronian era, it is fragmented into three domains. The literature does not appear to address this fragmentation yet. Table 2 summarises the subfindings and shows the key aspects of this fragmentation. Various issues can be discussed in consideration of this fragmentation.

Issue 1 is that education in the coronian epoch is a hybrid of three domains (domestic, digital and political). The departure of education from a school setting has resulted in the disappearance (and, therefore, destruction) of such a historically persistent realm. Beyond naivety, to ignore this destruction is to take flight from the social reality that needs to be understood and critically analysed. On analytical grounds, as education enters more domains and, therefore, its domains spread, more actors become involved, turning the education system into a “power play” (interviewee) where the new actors hunt for power, influence and profit. It seems sensible to describe coronian education as a political “battlefield where everyone “shoots” everyone in the head” (interviewee). This battle manifests itself on social media where various bouts can be watched. It is here one can witness the collision of actors from the domestic, digital and political domains.

Issue 2 is that, although academic investigations into pre-coronian education required researchers to approach merely one domain (the school setting), investigating coronian education necessitates approaching three domains (domestic, digital and political), thus distracting and draining researchers. The introduction of education into novel, vast and complex domains and the rise of new and diverse actors make it challenging for researchers to identify these new actors, listen to all their interests and record their voices. This is why this project ended up recruiting many data collectors and conducting many interviews and observations, as we tried to cast as wide a net as possible into the multifaceted network of these new actors. To capture the views of these diverse actors, the strategy of maximum variation sampling was applied by approaching various groups.

Issue 3 is that, although researching education in its digital and political domains is feasible, it is challenging to carry out such research in its domestic domain. This is because conducting cultural research on education at home means that enquiries have to invade the private spaces of homes. To circumvent this challenge in the current study, an army of data collectors was established on the condition that each performs research within their own private (and semi-private) domains (i.e., their relatives and friends). Considering that Arabs tend to have large families and create continuously grander circles of friends, each data collector managed to find a wealth of participants. Those being researched, as relatives and friends of the data collectors, were motivated to speak openly, freely and comfortably, which is uncommon in research on Arabs due to the power of social surveillance. By virtue of family solidarity, whereby Arab family members help one another succeed at any cost, the data collectors’ family members voluntarily turned themselves into unofficial data collectors, conducting interviews and observations and sharing data with the official data collectors. Entire families transformed themselves into active "beehives" that proactively collected data and spread a culture of cultural investigation within families. Thanks to the social value of family solidarity, the response rate for the current study was almost 90%, subverting the recorded average of a 50% response rate in qualitative enquiries [29]. Some may raise the concern that the data were collected from batches of family members and friends, which can create problems because not all data points were independent of each other, as individuals in the same social groups are more likely to have the same opinions. If this is the case, the batches should be combined into single observations to more accurately represent the distribution of different sentiments. Yet, because the phenomenon of coronian education is culturally new and unique, the perspectives were found to vary even within one family. This variation is partly because family members were influenced not by themselves but, more essentially, by their exposure to social media sites and the various perspectives on these sites.

Issue 4 is that once education leaves its “territory” (i.e., the school building) and enters various other territories, it loses its quality. Expressed bluntly, there arguably existed substantially limited actual learning during the pandemic. A witty post said, “If distance intercourse results in children, distance education results in educated children”. What existed, however, was merely a façade of learning, which gave rise to a coronian learning façade. To illustrate the point, although the students’ educational engagement with and acquisition of knowledge was low, a façade of enhanced attendance and improved grades for assignments and examinations emerged because their parents attended classes on behalf of the children and took examinations for them. Therefore, coronian education masquerades as education and hides the reality of minimal significant learning. It is *zy altabl manfukh ealaa alfadi* (like a balloon—large, with no core). Such limited learning outcomes emerged by dint of the managerial ego to ensure the historical continuity of the education system by any means, despite its ineffectiveness. It arose from the sudden, defensive and unplanned transformation of all the scattered education components, from face-to-face education to distance education.

Issue 5 is that education departed from the school domain and entered three alternative domains (domestic, digital and political). Some may wonder what would happen when or if education returns to its traditional home. It can be argued that the influence of these three alternative domains will simply and passively vanish completely and that the actors that arose due to the introduction of these domains will effortlessly exit stage left, never to be seen again. Instead, the case may be that there will be a severe power struggle between the school domain and these three new domains. Eliminating the influence of these alternative domains will necessitate exterminating all their actors, and this is when the real struggle will begin. Watching, documenting and analysing the return of education to where it belongs—the school building—is worthy of detailed investigation. This is a conjecture and/or prediction about the future that cannot be studied (yet), but it is a direction for future studies should certain possible future events occur.

To sum up, this study showed that the pandemic caused a fundamental change in the philosophy of education through its principles, values and people’s perceptions, thus announcing the arrival of an original type of education (coronian education). From a mere health phenomenon, the virus became an influencer over education (and other aspects of human life and civilisation). A key difference between pre-coronian and coronian education is that physicality was the core of the former, whereas digitality is the core of the latter. In pre-coronian education, calls were consistently made for the integration of technology into education. In coronian education, education was integrated into technology. Coronian education forced face-to-face education to a stop, which made the education system lose its historical, antique sense of tangibility. It challenged the conventional temporal structure of education and made some stakeholders save or lose time. It was broadcast from homes, compromising the privacy of families’, students’ and teachers’ residences. It modified the power relations between the two genders and among schools, students and families. It eliminated the existing actors and welcomed new stakeholders, and it reshaped the existing power relations and gave rise to new ones.

A key concluding remark concerns the finding that a large number of social initiatives have been taken to ease the experience of coronian education. This generous social support is understood as being rooted in the high value that Arabs place on “ata” (*translation*: givingness). Notwithstanding the multitude of values Arabs possess, all such values feed into and are fed by the one value of givingness [30]; givingness "attracts, gathers and leads to almost all Arab moral values" [31], p. 10. Culturally speaking, one’s givingness speaks of one’s courage, nobility, strength and chivalry [32,33]. To maintain this givingness-based social reputation, institutions and individuals invest substantial time and effort into publicly exhibiting a high level of givingness [34]. They exploit any opportunity (here, the pandemic) to put on a display of this quality [35]. This quality is exercised not necessarily for the sake of humanity, but rather because it is “the highway to social sovereignty, superiority, visibility, respect, prestige, power and honour” [36], pp. 156–157, which “enables one to rise in the ladder of glory and hierarchies” [37], p. 14. In short, Arab society is structured around the concept of “power and give” (cf. [38]’s theory of “power and love”).

Another key concluding remark is related to the finding that many mothers have been taking their children’s examinations in their place. This finding is culturally more complicated than it seems. In Arab culture, mothers typically play two roles in relation to their children. The first role is an instructive, moral one; for example, instructing children to act honestly. The second role is an assistive, comforting one; for example, making children feel supported and comforted. Coronian education has destabilised these roles and forced mothers to prioritise one role over the other. In the first approach, a mother takes her children’s examinations, providing substantive support to her children but, in so doing, instilling in them the utility of dishonesty. In the second approach, a mother refuses to take her children’s examinations; although fulfilling her role as a moral instructor, she risks her children feeling that she has withdrawn her support or does not support them unconditionally. Under circumstances where they must choose between these two approaches, many mothers appear to give more weight to their assistive, comforting role than their instructive, moral role.

An additional concluding remark concerns the finding that coronian education has changed students’ perception of how they should be assessed and graded. Coronian-educated students feel a sense of entitlement to good grades. They believe that their performance has suffered due to the pandemic, and teachers should take this into consideration. There has also been a shift in teachers’ perception of how students should be evaluated and graded. Some teachers have given students lower grades during the pandemic because the online nature of coronian education reduces students’ hardship (as they can “easily” and “comfortably” receive education at home through technology). For these teachers, *al ajer aala qader al mashaqah* (Arab saying: “rewards should be based on hardship, not quality and outcome”) has guided their grading. Moreover, some teachers have designed examinations not for the students but for their mothers, as they hold the assumption that mothers, not the students themselves, will be taking the examination. Therefore, students who refuse to cheat become victims of the system. 

One more concluding remark concerns the finding that, on social media, social campaigns and bottom-up e-votes have sprouted up over coronian school procedures and regulations and, moreover, over social norms and values. Such campaigns, notably, did not exist in pre-coronian education. One reason is that the pre-coronian education system was widely regarded as a stable system that had developed over generations. As a result, people did not dare to question this system. In contrast, the coronian education system is a wholly modern phenomenon, and the current generation feels a sense of belonging and ownership towards it. Accordingly, they feel entitled to negotiate how its procedures should be formulated and delineated. The problem, however, arises due to the fact that Arab society is not composed of education specialists, and Arab social media is dominated by emotions, cynicism and a lack of constructive critical thinking. There is, therefore, the risk that social participation in building an educational system will lead this system not in a positive and constructive direction, but to the “edge of the abyss” (interviewee).

This research raised various issues related to Arab culture. However, it would be academically beneficial to examine these issues in relation to other non-Arab cultures. One such issue is the application of witticisms as a data collection method in non-Arab contexts. Another issue is an enquiry into who teaches the students at home in non-Arab coronian education and whether parents do the homework and take examinations on behalf of their children. This is in addition to examining the potential influence of coronian education over disputes between parents and children, between teachers and parents, and between students and teachers in non-Arab settings. An additional issue is the extent to which non-Arab coronian education has compromised students’ and teachers’ privacy. The influence of education on students’ discipline and the influence of home-based distractions on students’ concentration during coronian education can be additional issues. It is also worth investigating whether education in non-Arab contexts became culturally and societally less valued once it became virtual during the pandemic. Another matter that can be investigated is the degree to which coronian education has diminished the conventional rituality of classrooms and schools, manifested in boards, board pens, chairs, tables, projectors, bells, lesson lengths, examination times, the school day’s length, etc. It would also be interesting to enquire into the locations (e.g., stables, markets, mosques, boats, hotel rooms, walkways, bicycle paths and beauty salons) in which non-Arab students have attended coronian education classes. Whether non-Arab coronian education has influenced students’ physical activities and institutions’ and parents’ savings related to electricity, water, maintenance, computers, school clothing, supplies and meals also deserves academic examination.

## Figures and Tables

**Table 1:** Authoring Group Formation and Data Collection.

**Starting Point**
The initiator of "The Arab Research Group for Education during the Pandemic" acts as the project coordinator.
↓
**Making a Call for Membership**
The coordinator announces the opening of candidacies for membership of the group via social networks.
↓
**Collecting Nominations for Membership**
Arab academics nominate themselves for group membership by filling out a form. The form consists of the following question: "From your perspective, please list 10 differences between education before and during the pandemic." That is, the form is more like a test that seeks to judge the academic quality of the nominated through how good their answers are to the question in the form.
↓
**Screening Nominations for Membership**
The coordinator forms a committee (consisting of three academics who have published at least 10 Clarivate-indexed articles) that evaluates the candidates’ responses to the form. The committee selects for group membership those whose responses are remarkable. The committee is also concerned with enacting the terms and conditions for this group.
↓
**Setting Terms and Conditions for the Group**
The formed committee enacts the terms and conditions for the group dynamics.
↓
**Announcement of Admitted Candidates**
The admitted candidates are informed, and the group begins its work directly and communicates through WhatsApp.
↓
**Collecting Data**
Each member conducts one interview, one observation of offline life and one observation of online life. They transfer the data to a "research e-card" on a daily basis.
↓
**Cleaning Data**
The coordinator forms a data-cleaning team of 11 people whose role is to clean the data entered into the research e-cards.
↓
**Entering Data into NVivo**
The coordinator forms a technical team of 11 technicians whose role is to enter the data into NVivo.
↓
**Analysing Data**
The coordinator forms a data-analysis team of 11 people whose role is to analyse the research data entered into NVivo.
↓
**Generating Findings**
The data-analysis team sends the findings emerging from their analysis to the coordinator.
↓
**Dividing the Team into Subgroups**
Although these data are collected by a large group, they are made publishable by using subgroups. A significant collaboration enables the collection of sizeable data sets, from which each member or small subgroup can reap benefits independently of the group. In this case, the group members collaborate for the benefit of each other.

**Table 2:** Summary of Subfindings.

Domestic Domain	Digital Domain	Political Domain
**Added Responsibilities**	**Reduced Tangibility**	**Participatoriness**
In coronian education, family members are the ones teaching their children, yet they also take their children’s examinations in their place.	Coronian education entails an absence of physical attendance, both of students and staff.	Coronian students, parents and teachers turn to social media to exert pressure on school management.
**Related Existing Study**	**Related Existing Study**	**Related Existing Study**
Ref. [20] highlighted the role of mothers in students’ unethical behaviour during the pandemic.	Ref. [21] confirmed attendance of students and staff has gone online during the pandemic.	No existing study could be found on this issue.
**Fostered Tension**	**Lessened Rituality**	**Reshaped Relations**
Coronian education ushers in enhanced forms of disputes between parents and children, between teachers and parents, and between children and teachers.	Coronian education considerably diminishes the conventional rituality of classrooms and schools (e.g., boards, board pens, chairs, tables, projectors and bells) and destabilises “divine temporal configurations” (e.g., lesson lengths, examination times, and the school day’s length).	Coronian education reshapes the power relations between the two genders.
**Related Existing Study**	**Related Existing Study**	**Related Existing Study**
Ref. [22] drew attention to a similar (yet not discussed in the current research) type of dispute: disputes among parents themselves.	Ref. [23] shed light on the profound challenge that the pandemic has posed to existing temporal configurations of education.	Ref. [24] elaborated on the impact of the pandemic on gendered power relations in gender-separated educational environments.
**Compromised Privacy**	**Mobility**	**Institutional Fabrication**
Under coronian education, more actors (essentially anyone with access to a student’s account) can monitor students’, as well as teachers’, performance.	Coronian education enables schooling to take place anywhere.	Many Arab institutions dedicate their energies to winning awards for their effective management of coronian education.
**Related Existing Study**	**Related Existing Study**	**Related Existing Study**
No existing study could be found on this issue.	Ref. [25] singled out the changes that have taken place owing to the enhanced mobility of education caused by the pandemic.	Ref. [26] reported on such awards, yet do not discuss them in any detail.
**Enhanced Distraction**	**Unhealthiness**	**Social Support**
Coronian students’ ability to concentrate is diminished, as the Arab household is conventionally beset by distractions.	In coronian education, physical activity is significantly reduced because from-home education is not conducive to the sports that students previously practised in pre-coronian education.	Many social initiatives are taken to ease the experience of coronian education in addition to efforts to spread a sense of positivity during coronian education.
**Related Existing Study**	**Related Existing Study**	**Related Existing Study**
Ref. [27] discussed the distractions at home that Arab students have encountered during the pandemic.	Ref. [28] explored the impact of the pandemic on students’ physical activity.	No existing study could be found on this issue.

## Data Availability

The data are confidential and, therefore, not available to the public.
